# Comparison of 3 Infrared Thermal Detection Systems and Self-Report for Mass Fever Screening

**DOI:** 10.3201/eid1611.100703

**Published:** 2010-11

**Authors:** An V. Nguyen, Nicole J. Cohen, Harvey Lipman, Clive M. Brown, Noelle-Angelique Molinari, William L. Jackson, Hannah Kirking, Paige Szymanowski, Todd W. Wilson, Bisan A. Salhi, Rebecca R. Roberts, David W. Stryker, Daniel B. Fishbein

**Affiliations:** Author affiliations: Centers for Disease Control and Prevention, Atlanta, Georgia, USA (A.V. Nguyen, N.J. Cohen, H. Lipman, C.M. Brown, N.A. Molinari, W.L. Jackson, H. Kirking, P. Szymanowski, T.W. Wilson, D.B. Fishbein);; Council of State and Territorial Epidemiologists, Atlanta (A.V. Nguyen);; Emory University, Atlanta (P. Szymanowski, B.A. Salhi);; John H. Stroger, Jr. Hospital of Cook County, Chicago, Illinois, USA (R.R. Roberts);; Presbyterian Healthcare Services, Albuquerque, New Mexico, USA (D.W. Stryker)

**Keywords:** bioterrorism and preparedness, mass screening, infrared thermal detection systems, self-reported fever, research, *Suggested citation for this article*: Nguyen AV, Cohen NJ, Lipman H, Brown CM, Molinari N-A, Jackson WL, et al. Comparison of 3 infrared thermal detection systems and self-report for mass fever screening. Emerg Infect Dis [serial on the Internet]. 2010 Nov [*date cited*]. http://dx.doi.org/10.3201/eid1611.100703

## Abstract

Despite limited evidence regarding their utility, infrared thermal detection systems (ITDS) are increasingly being used for mass fever detection. We compared temperature measurements for 3 ITDS (FLIR ThermoVision A20M [FLIR Systems Inc., Boston, MA, USA], OptoTherm Thermoscreen [OptoTherm Thermal Imaging Systems and Infrared Cameras Inc., Sewickley, PA, USA], and Wahl Fever Alert Imager HSI2000S [Wahl Instruments Inc., Asheville, NC, USA]) with oral temperatures (>100°F = confirmed fever) and self-reported fever. Of 2,873 patients enrolled, 476 (16.6%) reported a fever, and 64 (2.2%) had a confirmed fever. Self-reported fever had a sensitivity of 75.0%, specificity 84.7%, and positive predictive value 10.1%. At optimal cutoff values for detecting fever, temperature measurements by OptoTherm and FLIR had greater sensitivity (91.0% and 90.0%, respectively) and specificity (86.0% and 80.0%, respectively) than did self-reports. Correlations between ITDS and oral temperatures were similar for OptoTherm (ρ = 0.43) and FLIR (ρ = 0.42) but significantly lower for Wahl (ρ = 0.14; p<0.001). When compared with oral temperatures, 2 systems (OptoTherm and FLIR) were reasonably accurate for detecting fever and predicted fever better than self-reports.

## Abstract 2

In a hospital setting, the systems had reasonable utility for fever detection.

Advancements in transportation coupled with the growth and movement of human populations enable efficient transport of infectious diseases almost anywhere in the world within 24 hours (*1*). This recognition has prompted the evaluation of rapid mass screening methods to delay the importation of infection into healthcare settings, communities, and countries *(**1**–**4**)*. Because fever is a common indicator of many infectious diseases, the rapid identification of fever is a major component of screening efforts. Such screening was used by many countries during the severe acute respiratory syndrome outbreak in 2003 and the influenza A pandemic (H1N1) 2009 outbreak (*2*,*3*,*5*–*8*). Despite widespread implementation of fever screening, its value for detecting highly communicable diseases has mainly been established through mathematical modeling rather than through studies in humans (*9*,*10*).

One approach to fever screening is to simply ask persons if they have a fever. In healthcare settings, this information is routinely obtained in the chief complaint or review of symptoms and in some situations by querying persons as they enter the facility (*11*). In travel settings, many countries have used a written health declaration to screen travelers arriving at international ports of entry (*2*). However, limited information exists on the accuracy of self-reported fever, which is biased by its subjective nature and reliance on travelers’ awareness of fever status and willingness to report (*12*,*13*). Indeed, a clinical trial suggested that traditional thermometry is superior to self-reported fever for identifying patients with seasonal influenza (*14*). However, traditional thermometry methods are time-consuming and require close contact with potentially infectious patients.

Infrared thermal detection systems (ITDS) offer a potentially useful alternative to contact thermometry. This technology was used for fever screening at hospitals, airports, and other mass transit sites during the severe acute respiratory syndrome and influenza A pandemic (H1N1) 2009 outbreaks (*2*,*3*,*5*–*8*,*15*). ITDS appeared to enable early detection of febrile persons entering healthcare facilities, where the undetected introduction of communicable diseases can lead to outbreaks among patients and staff (*5*,*16*–*18*).

Although ITDS have the potential to serve as rapid, noninvasive screening tools for detecting febrile persons, previous studies provide conflicting information about their utility for mass fever screening (*15*,*16*,*19*–*25*). In addition, there are few published comparisons of the efficacy of different ITDS and their suitability for mass fever screening (*19*). Finally, no studies on the relative accuracy of self-reported fever and ITDS for fever screening or the value of combining these 2 methods have been published. These questions and the potential need to rapidly screen for fever during an emerging pandemic prompted us to conduct this study to validate different ITDS temperatures and self-reported fevers with oral temperatures.

## Methods

### Study Setting

A cross-sectional study comparing 3 ITDS was conducted in 3 urban tertiary-care hospital emergency departments in the United States: Albuquerque, New Mexico; Atlanta, Georgia; and Chicago, Illinois. Emergency departments were selected as the evaluation setting because of a potential high prevalence of fever compared with its prevalence in healthy populations and the routine measurement of each patient’s oral temperature. The 3 hospitals were selected because of their estimated patient volume of >200 patients per day.

### Human Subject Research Protections

The study was approved by the Institutional Review Board (IRB) of the Centers for Disease Control and Prevention (CDC) and the IRBs of the hospitals in Atlanta and Chicago. The Albuquerque hospital’s IRB reviewed the protocol but deferred to CDC’s IRB for approval.

### Device Selection

ITDS were selected for evaluation through a competitive bidding process. Selection criteria included specifications suitable for fever screening: view field captures human heights (0.5–2.5 meters), temperature discrimination <0.2°C, smallest available sensor temperature range encompassing human temperatures (–40°C to 120°C), tripod/stationary mount, operational distance >2 meters, internal/external calibration standards, temperature capture time <1 second, and price <$25,000. Of 6 devices submitted to CDC, 3 met the above criteria and were selected for testing: the FLIR ThermoVision A20M (FLIR Systems Inc., Boston, MA, USA), the OptoTherm Thermoscreen (OptoTherm Thermal Imaging Systems and Infrared Cameras Inc., Sewickley, PA, USA), and the Wahl Fever Alert Imager HSI2000S (Wahl Instruments Inc., Asheville, NC, USA). Manufacturers provided training and consultation on the assembly and operation of the ITDS per company practices but were otherwise uninvolved in the study.

### Participants and Eligibility

Adults (>18 years of age) were recruited consecutively among patients who sought care at the emergency departments of 1 hospital in each city: Chicago (September 15–29, 2008), Atlanta (October 6–24, 2008), and Albuquerque (February 17–26, 2009). Patients were approached after they had been registered in the emergency department from 7:00 am to 11:00 pm, 7 days per week, at all 3 sites and were enrolled in the study if they were willing to participate and gave verbal consent. Patients who were nonambulatory, mentally incompetent, arrested or incarcerated, <18 years of age, or required immediate medical attention were excluded from the study. Pregnant women were excluded in Chicago and Atlanta at the request of the hospitals’ IRBs.

### Sample Size

We estimated that 61 febrile patients were necessary to evaluate the sensitivity of ITDS for fever detection (assumed to be 80% from previous research) to within ±10% with 95% confidence. With an estimated fever prevalence of 2% among a population of patients at emergency departments, a total sample size of ≈3,000 patients was needed for the study.

### Temperature Measurements

The 3 ITDS were positioned at the optimal distance (2–3 m) from each participant as recommended by the manufacturers. Each ITDS camera field of view was preset to capture the patient’s face and neck. Participants were asked to remove eyeglasses and hats and instructed to stand facing the cameras until temperature measurements from all 3 devices had been recorded.

To account for ambient temperature, the Wahl device was manually calibrated on each morning before data collection, per manufacturer recommendation. In Albuquerque, where room temperatures varied during the day, the Wahl was additionally calibrated after noticeable changes in ambient temperature. The OptoTherm and FLIR have automated calibration systems to adjust for ambient conditions, diurnal variations in temperature, and thermal drift and therefore did not require manual calibration.

Unadjusted skin temperatures detected by ITDS were included in the analysis to enable direct comparison with oral temperature measurements. The FLIR and Wahl cameras did not display fixed temperature readings but rather readings that fluctuated by tenth of a degree increments. For these 2 cameras, operators recorded the highest temperature displayed for each person. Measurements recorded by the FLIR during periods when the camera was not properly focused were excluded from the analysis.

Oral temperatures were measured by clinical staff using a DinaMap ProCare digital thermometer (General Electric Company, Freiburg, Germany) in Albuquerque and Atlanta and a Welch Allyn SureTemp Plus 692 Electronic Thermometer (Welch Allyn Inc, San Diego, CA, USA) in Chicago, per each hospital’s established patient care standard. ITDS temperature measurements were taken either immediately after (Chicago and Atlanta) or just before (Albuquerque) each oral measurement. Confirmed fever was defined as an oral temperature >100°F (>37.8°C). Room temperatures were recorded hourly by using a standard digital room thermometer.

### Patient Self-Reports

Upon enrollment, patients were asked, “Do you feel like you have a fever now?” (self-reported fever) and whether they had taken medication for pain or fever (analgesic or antipyretic drugs) in the previous 8 hours. When needed, patients were given examples of trade and generic names of common antipyretic drugs. Their responses, along with each patient’s age and sex, date, and time of temperature measurement were recorded.

### Data Analysis

Symptom questionnaire responses, oral temperature measurements, and ITDS-recorded data were entered into an Excel (Microsoft Corp., Redmond, WA, USA) database and analyzed by using SAS Version 9.2 (SAS Institute Inc, Cary, NC, USA). Patient responses of “Don’t know” to the question, “Do you feel like you have a fever now?” were analyzed as “No.” ITDS and oral temperature measurements were compared by using descriptive statistics and bivariate analysis (χ^2^ tests, *t* tests, and correlations). Generalized linear modeling was used to investigate the effects of covariates and potential confounders (age, sex, recent antipyretic use, study site, self-reported fever, time of day, and room temperature) on temperature measurements and to identify factors that influenced the difference between oral and ITDS temperature measurements, given site-specific effects.

Sensitivity (the proportion of those with confirmed fever who were identified as febrile by ITDS) and specificity (the proportion of those without confirmed fever who were identified as nonfebrile by ITDS) were calculated and used to plot the receiver operating characteristic (ROC) curves for all possible fever temperature thresholds on each ITDS. Optimal ITDS fever thresholds were defined as the temperature that yielded the highest combined sensitivity and specificity for fever detection for each device as determined by the ROC curves. Positive predictive value (PPV), the proportion of patients identified as febrile by ITDS who had a confirmed fever by oral temperature, was compared with self-report. The accuracies (sum of sensitivity and specificity) of the following 3 fever screening methods were compared by using oral thermometry as reference: 1) self-reported fever, 2) ITDS at optimal fever detection threshold, and 3) combination of ITDS and self-reported fever with a positive result on either method considered a fever.

## Results

Of 3,345 eligible patients, we enrolled a total of 2,873 (85.9%): 1,511 (52.6%) in Chicago, 1,040 (36.2%) in Atlanta, and 322 (11.2%) in Albuquerque. The remaining 472 (14.1%) patients refused to participate. Men accounted for 1,514 (52.7%) participants; the mean age was 42 years (range 18–92 years). The mean oral temperature was 97.9°F (range 92.8°F–104.4°F); 64 (2.2%) patients had confirmed fever, including 48 (10.1%) of 476 patients reporting fever. Antipyretic or analgesic drug use within 8 hours was reported by 1,121 (39.0%) patients, including 225 (45.8%) who self-reported fever and 39 (60.9%) who had confirmed fever.

**Figure 1:**
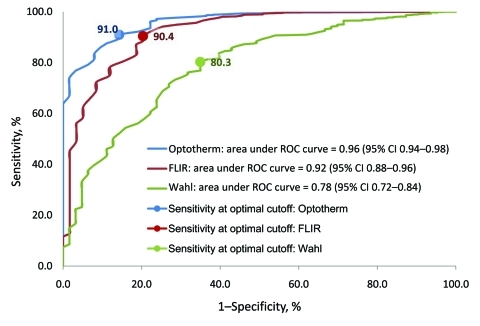
Receiver operating characteristic (ROC) curves of 3 infrared thermal detection systems (ITDS) for detecting fever (oral temperature >100°F): FLIR ThermoVision A20M (FLIR Systems Inc., Boston, MA, USA), OptoTherm Thermoscreen (OptoTherm Thermal Imaging Systems and Infrared Cameras Inc., Sewickley, PA, USA), and Wahl Fever Alert Imager HSI2000S (Wahl Instruments Inc., Asheville, NC, USA). CI, confidence interval.

**Figure 2:**
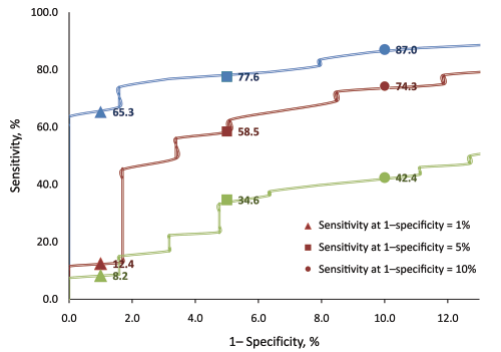
Enhanced view of receiver operating characteristic curves of 3 infrared thermal detection systems for detecting fever (oral temperature >100°F) showing sensitivities at false-positive rates (FPR) of 1%, 5%, and 10%. Red, FLIR ThermoVision A20M (FLIR Systems Inc., Boston, MA, USA); blue, OptoTherm Thermoscreen (OptoTherm Thermal Imaging Systems and Infrared Cameras Inc., Sewickley, PA, USA); and green, Wahl Fever Alert Imager HSI2000S (Wahl Instruments Inc., Asheville, NC, USA).

Correlations of ITDS and oral temperatures were similar for OptoTherm (ρ = 0.43) and FLIR (ρ = 0.42) but significantly lower for Wahl (ρ = 0.14; p<0.001). The areas under the ROC curves (AUC) for OptoTherm (96.0%) and FLIR (92.0%) were not significantly different but were significantly greater than the AUC of Wahl (78.2%; p<0.001; Figure 1). At their respective optimal threshold temperatures, sensitivities of fever detection of the 3 ITDS were not significantly different from each other, but specificities and PPVs of OptoTherm and FLIR were significantly higher than those of Wahl (Table 1; p<0.001). At fixed specificities, the sensitivities of each ITDS varied (Figure 2).

**Table 1:** Comparisons of 3 infrared thermal detection system results and self-reported fever with oral temperature among patients in 3 emergency departments, USA, 2008–2009*

Characteristics	OptoTherm, n = 2,507 patients	FLIR, n = 2,515 patients	Wahl, n = 2,061 patients	Self-reported fever, n = 2,389 patients
Mean temperature, °F (SD)	94.3 (1.26)	95.7 (1.38)	89.4 (2.56)	–
Optimal fever threshold, °F	95.3	96.4	89.3	–
Fever (oral temperature >100°F)				
No. (%) identified as febrile by each method	275 (11.0)	247 (9.8)	577 (28.0)	404 (16.9)
Sensitivity (95% CI)	91.0 (85.0–97.0)	90.0 (84.0–97.0)	80.0 (76.0–85.0)	75.0 (64.4–85.6)
Specificity (95% CI)	86.0 (81.0–90.0)	80.0 (76.0–84.0)	65.0 (61.0–69.0)	84.7 (83.4–86.1)
Positive predictive value (95% C)	17.9 (13.6–22.2)	18.4 (13.7–23.0)	5.7 (4.1–7.3)	10.1 (7.4–12.8)
Negative predictive value (95% CI)	99.6 (99.3–99.8)	99.5 (99.1–99.7)	99.1 (98.6–99.5)	99.3 (98.9–99.6)
Febrile by either ITDS or self-report				
No. (%) identified as febrile by each method	597 (23.8)	586 (23.3)	793 (38.5)	–
Sensitivity (95% CI)	93.8 (87.8–99.7)	89.1 (81.4–96.7)	93.8 (87.8–99.7)	–
Specificity (95% CI)	78.0 (76.4–79.5)	78.4 (76.9–80.0)	63.3 (61.6–65.1)	–
Positive predictive value (95% CI)	9.0 (6.9–11.2)	8.8 (6.8–11.3)	5.6 (4.3–7.1)	–
Negative predictive value (95% CI)	99.8 (99.5–99.9)	99.7 (99.3–99.9)	99.8 (99.4–99.9)	–

*OptoTherm Thermoscreen (OptoTherm Thermal Imaging Systems and Infrared Cameras Inc., Sewickley, PA, USA), FLIR ThermoVision A20M (FLIR Systems Inc., Boston, MA, USA), and Wahl Fever Alert Imager HIS2000S (Wahl Instruments Inc., Asheville, NC, USA). CI, confidence interval; ITDS, infrared thermal detection system.

Compared with oral thermometry, sensitivity for self-reported fever was 75%, specificity was 84.7%, and PPV was 10.1%. Sensitivities of the 3 ITDS at their respective optimal thresholds did not differ significantly from that of self-reported fever (Table 1). However, specificities and PPVs of OptoTherm and FLIR at optimal thresholds were significantly greater than those of self-reported fever (p<0.001 for both comparisons), and specificity and PPV of Wahl were significantly lower than those of self-reported fever (p<0.001). The addition of self-report decreased the accuracy of fever detection at optimal thresholds for FLIR and OptoTherm (increase in sensitivity was less than decrease in specificity) but improved accuracy for Wahl with a greater increase in sensitivity than the decrease in specificity (Table 1). Conversely, adding OptoTherm or FLIR temperature measurements to self-reported fever increased accuracy, but adding Wahl temperature measurements decreased accuracy (Table 1).

Bivariate analyses revealed higher oral and ITDS temperatures among younger patients and later in the day (Table 2). Oral temperatures were higher in women, and ITDS temperature measurements were higher in men. ITDS temperature measurements increased with increasing room temperatures. Temperatures detected by oral thermometers, OptoTherm, and FLIR were higher in patients who reported recent antipyretic or analgesic drug use.

**Table 2:** Associations between temperature measurements by 3 infrared thermal detection systems and potential covariates, using bivariate analysis, among patients in 3 emergency departments, 2008–2009*

Characteristics	Oral thermometer, n = 2,873 patients	OptoTherm, n = 2,809 patients	FLIR, n = 2,314 patients	Wahl, n = 2,848 patients
Gender				
Male mean temperature, °F (SD)	97.85 (0.91)	94.36 (1.25)	95.77 (1.33)	89.52 (2.40)
Female mean temperature, °F (SD)	97.95 (0.87)	94.19 (1.27)	95.59 (1.40)	89.23 (2.73)
p value (*t* test)	0.002	<0.001	0.002	0.003
Age				
Correlation coefficient r	–0.12	–0.15	–0.10	–0.10
p value	<0.001	<0.001	<0.001	<0.001
Time of day				
Correlation coefficient r	0.08	0.24	0.19	0.27
p value	<0.001	<0.001	<0.001	<0.001
Antipyretic/analgesic use				
Yes (mean temperature °F) (SD)	97.96 (1.01)	94.39 (1.34)	95.76 (1.47)	89.47 (2.61)
No (mean temperature °F) (SD)	97.86 (0.81)	94.22 (1.20)	95.64 (1.32)	89.35 (2.54)
p value (*t* test)	0.003	<0.001	0.048	0.21
Room temperature				
Correlation coefficient r	0.01	0.19	0.19	0.19
p value	0.77	<0.001	<0.001	<0.001
Oral temperature				
Correlation coefficient r	–	0.43	0.42	0.14
p value	–	<0.001	<0.001	<0.001

*FLIR ThermoVision A20M (FLIR Systems Inc., Boston, MA, USA), OptoTherm Thermoscreen (OptoTherm Thermal Imaging Systems and Infrared Cameras Inc., Sewickley, PA, USA), and Wahl Fever Alert Imager HSI2000S (Wahl Instruments Inc., Asheville, NC, USA).

When we controlled for study site, multivariate analyses showed that 2 variables (sex and room temperature) were most strongly (p<0.001) associated with the size of the gap between oral and ITDS temperature measurements (Table 3). Smaller differences between ITDS and oral temperatures were found among men than among women. Differences between ITDS and oral temperatures became smaller with increasing room temperatures and as the day progressed (with the exception of FLIR). Site-specific effects indicated that, on average, differences between ITDS and oral temperatures were smaller among participants from Albuquerque and Atlanta than among those from Chicago. With the exception of Wahl measurements, the difference between ITDS and oral temperatures was greater in older patients. Differences between oral and OptoTherm temperatures tended to be smaller for those reporting antipyretic drug use.

**Table 3:** Association between measured temperature, difference, and covariates, general linear regression with site-specific fixed effects in 3 emergency departments, USA, 2008–2009

Characteristics	Oral, n = 1,865 patients	OptoTherm, n = 1,851 patients	Difference (oral–OptoTherm)	FLIR, n = 1,360 patients	Difference (oral–FLIR)	Wahl, n = 1,856 patients	Difference (oral–Wahl)
Intercept (SE)	98.220 (0.936)	15.027 (3.467)	14.426 (1.337)	15.777 (4.150)	14.309 (1.559)	13.160 (6.541)	21.769 (2.489)
Variable in model*							
Oral temperature	–	0.701†	–	0.693†	–	0.645†	–
Male sex	–0.055‡	0.254†	–0.271†	0.237†	–0.260†	0.501†	–0.522†
Age	0.011‡	–0.160§	0.019§	–0.029§	0.034§	–0.001‡	0.005‡
Age squared	–0.0002§	0.0001‡	–0.0002‡	0.0003§	–0.0004§	–0.002‡	0.0001‡
Site¶							
Albuquerque	–0.498†	0.915†	–1.061†	–0.214§	0.058‡	4.256†	–4.431†
Atlanta	–0.309†	0.514†	–0.603†	0.302§	–0.399†	0.043‡	–0.149‡
Time of day	0.104§	0.156§	–0.126§	0.131§	–0.100‡	0.352†	–0.315†
Time of day squared	–0.003§	–0.004§	0.003‡	–0.003‡	0.002‡	–0.008§	0.007§
Antipyretic use	0.106§	0.137§	–0.106§	0.118*	–0.086‡	0.075‡	–0.039‡
Room temperature	–0.010‡	0.133†	–0.137†	0.160†	–0.162†	0.131†	–0.135†
Self-reported fever (No)	0.432†	0.148§	0.022‡	0.149‡	0.003‡	–0.115‡	0.264§

*FLIR ThermoVision A20M (FLIR Systems Inc., Boston, MA, USA), OptoTherm Thermoscreen (OptoTherm Thermal Imaging Systems and Infrared Cameras Inc., Sewickley, PA, USA), and Wahl Fever Alert Imager HSI2000S (Wahl Instruments Inc., Asheville, NC, USA). Value of β coefficient (β) for each variable in the model is listed in the columns. †p<0.001. ‡Not significant (p>0.05). §p<0.05. ¶Referent site is Chicago.

## Discussion

Our evaluation of 3 ITDS in emergency department settings found that the FLIR and OptoTherm reliably identified elevated body temperatures. The high AUCs for these 2 systems suggest that they can differentiate between febrile and afebrile persons with relatively high sensitivity and specificity at an optimal fever cutoff. The relatively high correlation with oral temperature measurement also supports the utility of these 2 ITDS, which predicted fever better than self-reports of patients and more accurately alone than in combination with self-reported fever.

Our study is one of few that simultaneously examined the effects of multiple external and internal factors (age, sex, time of day, room temperature, and antipyretic drug use) on ITDS and oral temperature measurement accuracy. We found that ITDS and oral temperature measurements were strongly influenced by site and time of day, which may be a real effect or a result of variations in oral measurement techniques. The effects of age and time of day on body temperature found in this study have been well established by previous research (*26*–*28*). We observed strong associations between ITDS and room temperatures. Similar observations with room temperatures and extended exposure to hot or cold environments have been reported (*22*,*25*,*29*,*30*). The unexpected association between higher temperature measurements (oral and OptoTherm) and recent antipyretic drug use may result from patients with higher fevers taking antipyretic drugs, inadequate antipyretic drug dosage, or both. The finding that men had relatively higher ITDS measurements than women has not been previously reported and may be because of differences in facial hair, use of cosmetics, or subcutaneous fat composition (*31*). Similar associations across multiple ITDS underscore the strength of these findings. By controlling for these covariates, we were able to measure the relationship between ITDS and oral temperatures with greater precision.

Although the sensitivity, specificity, and AUC of the devices we tested were similar to those found in previous studies, we observed a higher correlation between ITDS temperature measurements and confirmatory temperature measurements (*15*,*16*,*19*–*25*). Several factors may have contributed to these differences. The higher correlation between ITDS and body temperatures reported here may be related to the use of oral temperature measurement as reference. Although oral temperature measurements better reflect core temperatures than infrared tympanometric measurements, most previous investigations of ITDS have used the latter as reference (*19*,*23*,*24*,*32*–*35*). The preference for oral temperatures as reference is supported by an evaluation of methods for measuring body temperature conducted by the American College of Critical Care Medicine and the Infectious Diseases Society of America; researchers found that rectal temperatures were the most accurate of the peripheral thermometry methods, followed by oral, tympanic, and axillary temperature measurements, respectively (*32*).

Many types of ITDS are available, ranging from inexpensive hand-held point-and-shoot devices with laser sighting to hand-held cameras with light-emitting diode displays, wall-mounted cameras, and portable cameras on tripods such as the ones used in this study (*19*,*23*,*29*). To maximize potential efficacy, we evaluated technically advanced ITDS that were recently developed for human temperature detection. Other studies used more basic systems and did not compare different devices. Although the costs of the OptoTherm and FLIR were comparable at $22,000 and $16,000 per system, respectively, the Wahl was relatively less expensive ($8,000). Testing 3 different models at various price ranges allowed us to demonstrate substantial differences among ITDS. These differences are likely to affect their sensitivity and utility for fever screening. The systems used in this study require the person to stand in front of the camera for ≈2–3 seconds to capture a temperature. Other differences, such as moving persons, could have further affected the sensitivity of ITDS for fever detection.

Although addition of a health declaration form would allow screening to also consider recent travel history, previous fever, and other symptoms or illness exposures, health declarations have variable compliance rates and depend on a person’s ability to understand questions and accurately assess symptoms as well as willingness to report (*12*,*13*,*36*,*37*). In our study, in which patients had no disincentive to report, we found that one fourth of febrile patients did not report having fever, which suggests true unawareness of fever among some persons. Only one tenth of those who reported having a fever were actually found to be febrile. Our results, therefore, probably underestimated the benefit of ITDS over self-reports of fever. In other settings, ill persons may be less likely to report symptoms for fear of adverse consequences such as travel delays, involuntary isolation of ill persons, or quarantine of exposed contacts. In settings such as travel sites (e.g., airports) and the workplace, ITDS could provide an objective means for the mass detection of fever as part of a comprehensive public health screening strategy because ITDS had greater accuracy than self-reports.

Mass health screening during a pandemic will certainly be influenced by several other factors, including perceived and actual pandemic severity, as well as the potential consequences of illness detection, either negative or positive, which can affect the sensitivity of screening that uses self-report. If being detected as febrile is perceived as harmful, travelers may hide their symptoms (*12*). Alternatively, during a pandemic with high mortality rates, incentives for reporting symptoms might be present, such as access to scarce antiviral medications and medical care. In both situations, a comprehensive screening approach may be necessary, which uses ITDS for fever screening and a health questionnaire to detect other symptoms or exposures that would increase specificity of the screening process. Finally, the usefulness of any infectious disease screening must take into account temperature fluctuations, use of antipyretic medications, transmission risks, prevalence of infections, and asymptomatic infections.

This study had several limitations. Measurement error resulting from variation in digital oral thermometer measurement and technique may have decreased the correlation between ITDS and oral temperature measurements (*38*). For FLIR and Wahl, varying readouts by different operators may have led to increased variability. This method, although necessary for direct temperature comparisons, may have decreased the accuracy of FLIR and Wahl. Use of alarm features as recommended by the manufacturers could minimize these differences but might lead to more false-positive results. In addition, unlike the other 2 devices, Wahl required calibration to ambient temperature once per day, but room temperatures varied within the day. We evaluated only systems submitted by manufacturers to the bid process, thus limiting the generalizability of our results to other devices.

To assess the sensitivity and specificity of different ITDS for fever detection and to determine their optimal thresholds, we validated each measurement by oral thermometry, which required a clinical setting. Thus, generalizability to settings such as airports and border crossings may be limited. Substantial delays to travelers and ethical concerns such as follow-up treatment made it impractical to conduct this study in an airport setting. In addition, although a few studies have examined screenings in airports, they confirmed temperature only in febrile persons, thus sensitivity and specificity of ITDS could not be established from such studies.

The sensitivity and specificity of screening by using ITDS are determined by the selected fever temperature cutoff, which tends to be 2–3 degrees lower than the standard fever threshold because of differences between skin and core temperatures. Increasing or decreasing sensitivity causes a reciprocal change in specificity. For example, lowering OptoTherm’s threshold from the optimal 95.7°F to 94.5°F would achieve almost 100% sensitivity but would reduce specificity to 63.6% and increase the false-positive rate to 36.4%; to reach near 100% specificity with the OptoTherm by using a cutoff of 100°F for ITDS, sensitivity decreases to 6.4%.

Maximizing accuracy by choosing the optimal cutoff with the highest sensitivity and specificity may not be practical in a real-world setting, considering the relative costs of false-positive and false-negative results. In settings where secondary evaluation is available or during a pandemic with high illness severity, ITDS temperature can be set at a lower cutoff to ensure fewer false negatives, each of which represents a potential public health threat. However, setting the cutoff to achieve very high sensitivity can result in many false positives, which could have adverse consequences to the population being screened (e.g., unnecessary travel delays, missed work) and increase the workload of staff who are conducting the screening. In settings where confirmatory testing may not be feasible or high costs may be associated with a false-positive result, a higher ITDS temperature cutoff may be preferable.
